# Gigantic laryngeal schwannoma: A case report with literature review

**DOI:** 10.1177/20363613241255669

**Published:** 2024-05-15

**Authors:** Daniel Nguyen, Nyein Nyein Htun, Cary Johnson

**Affiliations:** Department of Pathology and Laboratory Medicine, 14447 University of California, Irvine Medical Center, Orange, CA, USA

**Keywords:** Schwannoma, larynx, pathology, immunohistochemistry, nerve sheath tumor

## Abstract

Laryngeal schwannoma is a rare benign nerve sheath tumor that is slow growing. The diagnosis is made from a combination of clinical, radiological, and histopathological findings, and the main method of treatment is resection. We report a case of a 69-year-old presenting with a neck mass causing stridor, dysphagia, and orthopnea. CT of the neck showed an enhancing mass measuring 6.3 cm and extending superior to the larynx. Emergent tracheostomy and mass resection were performed, and histopathology and immunohistochemical findings were obtained from the specimen supporting schwannoma. In conclusion, while rare, schwannoma should always be considered as a differential diagnosis for a laryngeal mass. More studies are needed to assess the size and prognosis of the tumor.

## Introduction

Schwannoma is a mostly benign tumor of nerve sheath Schwann cells. While it commonly occurs in the head and neck region, other locations such as in the pelvic has been reported.^1^ Laryngeal schwannoma is rare. The diagnosis was made from a combination of clinical, radiological, and histopathological findings.^2^ Radiological diagnosis are essential in defining the morphological appearance of the tumor although histopathological examination is still needed for definite diagnosis.^1^ We present a case of a 69-year-old male with a slow-growing gigantic schwannoma originating from the larynx.

## Case presentation

**Figure 1.**
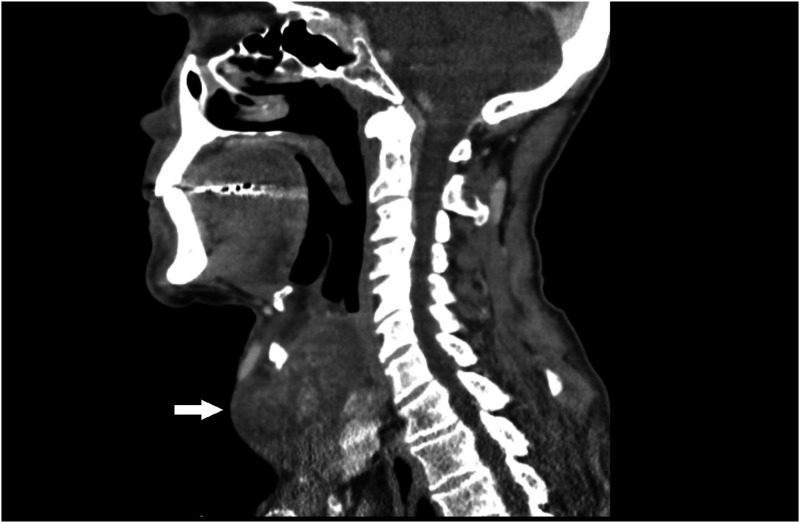
CT Soft Tissue of Neck to Show a Heterogeneous Enhancing Mass Measuring 6.3 x 5.8 x 4.1 cm (arrow pointed to the tumor).

The patient was a 69-year-old male from Nicaragua with no significant past medical history who presented to our hospital emergency room with a neck mass that had been progressively increasing in size for 2 years. He also experienced stridor, dysphagia and orthopnea. Physical examination was significant for a more than 10-cm central neck mass with a palpable thyroid notch but no palpable cricoid or trachea. CT Neck (Soft Tissue) revealed a heterogeneously enhancing mass with prominent vascularity abutting the superior aspect of the left thyroid gland and destroying the thyroid cartilage. The mass measured 6.3 × 5.8 × 4.1 cm by imaging and extended superiorly from the larynx (Figure 1). Emergent tracheostomy and neck exploration were performed to find a large, rubbery multilobulated mass in the prelaryngeal and pretracheal soft tissue that eroded most of the left thyroid cartilage into the paraglottic space, causing medialization and mass effects on the true and false vocal folds.

**Figure 2.**
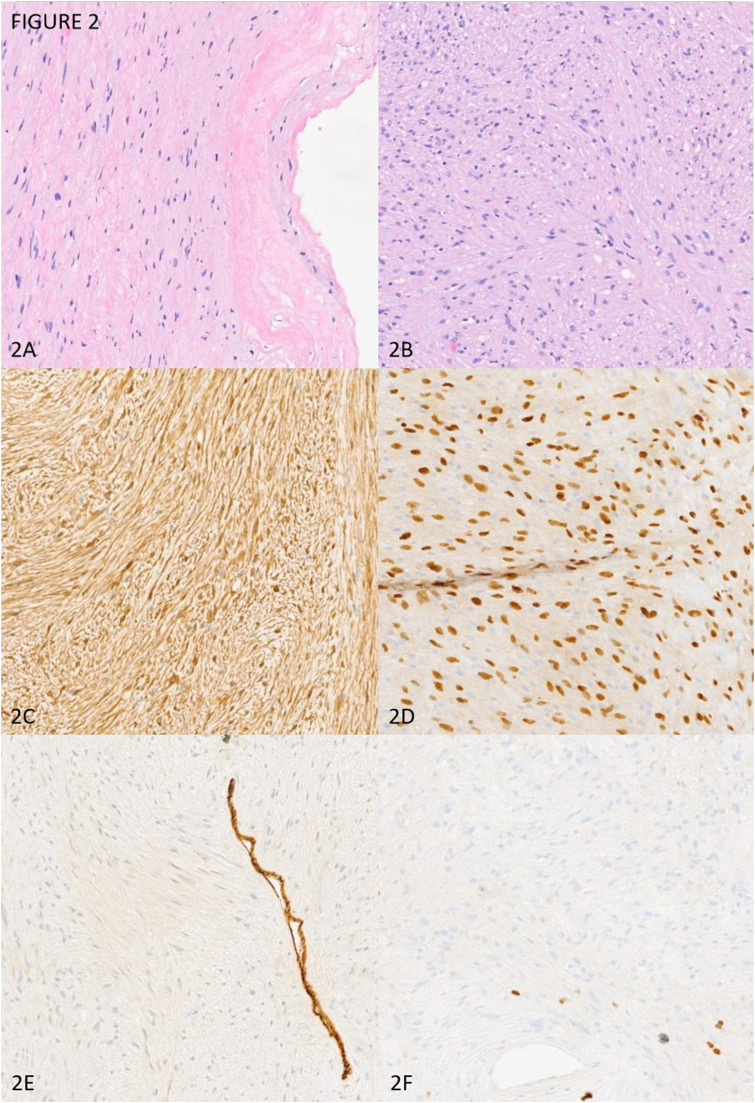
Microscopical And Immunohistochemical Profile of The Tumor; (A): Fibrous Capsule with Antoni B Pattern Area (10X); (B): Antoni A Pattern and Verocay Bodies (10X); (C): S100, strong and diffuse (10X); (D): SOX10, strong and diffuse (10X); (E): CD34, highlight on blood vessels (10X); (F): Ki-67, minimal less than 1% (10X).

The surgeons sent the frozen section specimen to our Pathology department, which found a low-grade spindle cell lesion. The mass and part of the larynx were resected en bloc and sent to Pathology for further work-up. Macroscopically, the lesion was an encapsulated soft tissue mass with a tan-white, homogenous cut-surface with the absence of an area of hemorrhage and necrosis. No gross laryngeal structure could be identified due to the erosion of the mass. Microscopically, the lesion was low-grade, spindled cell, and well-circumscribed. The tumor was composed of alternatively hypercellular Antoni A with nuclear palisading (Verocay bodies) and hypocellular Antoni B. Focal areas of hyalinization, nuclear atypia, and hemosiderin deposition were seen. No necrosis was identified. Fewer than one mitotic cell per 10 high-power fields was identified. By immunohistochemistry, the neoplastic cells stained strongly and diffusely positive for S100 and SOX-10. The neoplastic cells stained negatively or in nonspecific patterns for Pankeratin, DOG-1, Desmin, CD117 and CD34. The proliferation index Ki-67 was minimal (less than 1%). Our final diagnosis was a cellular peripheral nerve sheath tumor consistent with schwannoma (Figure 2).

After the procedure, the patient was stabilized and discharged from the hospital. The patient was followed up by Otolaryngology and Hematology-Oncology services as an outpatient. Four years after resection, the patient’s clinical course is going well without evidence of recurrence or metastasis.

## Discussion

Schwannoma, formerly known as neurinoma and neurilemmoma, is a type of benign neurogenic tumor. Approximately 45% of schwannomas are in the head and neck region, followed by the upper extremity (19%), lower extremity (13.5%) and trunk (8.5%).^3^ While schwannoma is common in the head and neck region, laryngeal schwannoma is rare, accounting for 0.1% of all benign laryngeal tumors.^4^ The size of the tumor has reported to vary from 5.8 to 0.3 cm in the greatest dimension.^5,6^ The tumor can be found in the false fold, true vocal fold, epiglottis and other laryngeal structures. Due to its location, some of the most common symptoms include dysphonia, hoarseness, dysphagia and dyspnea.^4^ The main method of treatment is surgical resection or laser excision.^2^

The tumor arises from the sheath of Schwann cells, which produces merlin protein and can expand and cause considerable injury to adjacent tissues.^7^ 90 percent of schwannomas occur in sporadic cases, with the remaining being linked to other syndromes, such as neurofibromatosis type 2.^8^ Schwannoma tumor progression has been linked with failure of Schwann cells to dedifferentiate upon nerve injury.^9^ Most lesions are solitary, slow-growing, painless masses with heterogeneous, degenerated and cystic cavitation on imaging findings.^10^ Histopathologically, laryngeal schwannomas are defined by hypercellular spindle cells with nuclei lined up in a palisading manner (Antoni A) and edematous regions of loosely arranged cells in degenerated background (Antoni B).^11^ The tumor is usually encapsulated. By immunohistochemistry, neoplastic cells are strongly and diffusely positive for SOX10 and S100.^12^ Another common differential diagnosis for schwannoma is neurofibroma, another type of nerve sheath tumor. However, schwannomas are smooth and encapsulated, while neurofibromas are more ill-defined and poorly circumscribed masses. SOX10 and S100 are strongly and diffusely expressed in schwannoma and are weaker in neurofibroma, while CD34 favors the latter.^13^

In our case, we favored the diagnosis of schwannoma due to the combination of radiological, histopathological and immunohistochemical findings. The tumor was well encapsulated and well defined. The appearance of both Antoni A with Verocay bodies and Antoni B patterns, diffuse positivity for S100 and SOX10, and a very low proliferation index are all characteristic of schwannoma. The tumor was gigantic, and it measured 6.3 cm and more than 10 cm of the gross specimen in the largest dimension by imaging, which is new for this type of tumor. However, this was a slow-growing tumor with no evidence of recurrence of metastasis, consistent with the characteristics of schwannoma.

## Conclusion

We report a case of a gigantic laryngeal schwannoma that grew slowly for more than 2 years in a 69-year-old patient. While laryngeal schwannoma is a rare tumor, it should always be included in a differential diagnosis. More studies are needed to assess the relationship between the size of a schwannoma and its prognosis.
